# High-resolution analysis for urinary DNA jagged ends

**DOI:** 10.1038/s41525-022-00285-1

**Published:** 2022-02-23

**Authors:** Tingting Xie, Guangya Wang, Spencer C. Ding, Wing-Shan Lee, Suk Hang Cheng, Rebecca W. Y. Chan, Ze Zhou, Mary-Jane L. Ma, Diana S. C. Han, Jeremy Y. C. Teoh, W. K. Jacky Lam, Peiyong Jiang, Rossa W. K. Chiu, K. C. Allen Chan, Y. M. Dennis Lo

**Affiliations:** 1 Li Ka Shing Institute of Health Sciences, The Chinese University of Hong Kong, Shatin, New Territories, Hong Kong SAR, China; 2 Department of Chemical Pathology, The Chinese University of Hong Kong, Prince of Wales Hospital, Shatin, New Territories, Hong Kong SAR, China; 3 Centre for Novostics, Hong Kong Science Park, Pak Shek Kok, New Territories, Hong Kong SAR, China; 4 S.H. Ho Urology Centre, Department of Surgery, The Chinese University of Hong Kong, Prince of Wales Hospital, Shatin, New Territories, Hong Kong SAR, China; 5 State Key Laboratory of Translational Oncology, The Chinese University of Hong Kong, Prince of Wales Hospital, Shatin, New Territories, Hong Kong SAR, China

**Keywords:** Tumour biomarkers, DNA sequencing

## Abstract

Single-stranded ends of double-stranded DNA (jagged ends) are more abundant in urinary DNA than in plasma DNA. However, the lengths of jagged ends in urinary DNA remained undetermined, as a previous method used for urinary DNA jagged end sequencing analysis (Jag-seq) relied on unmethylation at CpG sites, limiting the resolution. Here, we performed high-resolution Jag-seq analysis using methylation at non-CpG cytosine sites, allowing determination of exact length of jagged ends. The urinary DNA bore longer jagged ends (~26-nt) than plasma DNA (~17-nt). The jagged end length distribution displayed 10-nt periodicities in urinary DNA, which were much less observable in plasma DNA. Amplitude of the 10-nt periodicities increased in patients with renal cell carcinoma. Heparin treatment of urine diminished the 10-nt periodicities. The urinary DNA jagged ends often extended into nucleosomal cores, suggesting potential interactions with histones. This study has thus advanced our knowledge of jagged ends in urine DNA.

## Introduction

A number of recent efforts have been taken to explore the biological properties and clinical utilities of urinary cell-free DNA molecules^1,2^. Single-stranded overhangs of a double-stranded DNA fragment (i.e. jagged ends) were commonly present in plasma, which were possibly related to DNA fragmentation processes such as DNA nuclease activities^3,4^. In the plasma DNA pool, fetal DNA and tumoral DNA molecules were generally characterized with higher jaggedness compared with background DNA mainly of hematopoietic origin^4^.

In a recent study, the overall jaggedness of urinary DNA was found to be much higher than that of plasma DNA^1^. The jaggedness of urinary DNA decreased in patients with bladder cancer, compared with subjects without bladder cancer^1^. However, the previous study regarding urinary DNA jagged ends was based on the detection of unmethylated CpG sites introduced during the DNA end-repair process^1^. The analysis would limit the resolution of jagged end analysis of urinary DNA, because only 1% of dinucleotides in the human genome are CpG sites^4^. We reasoned that it would be informative to bring new mechanistic insights into jagged ends of urinary DNA using an approach with higher resolution by which the exact length of jagged ends could be determined. It was reported that the use of methylation signal at CH (H: A, C, or T) sites allowed the determination of exact length of jagged ends, on the basis of enhanced jagged end sequencing analysis (Jag-seq)^4^. Such methylation signals at CH sites were introduced during the DNA end-repair process with the use of methylated cytosines instead of unmethylated cytosines^4^.

**Fig. 1:**
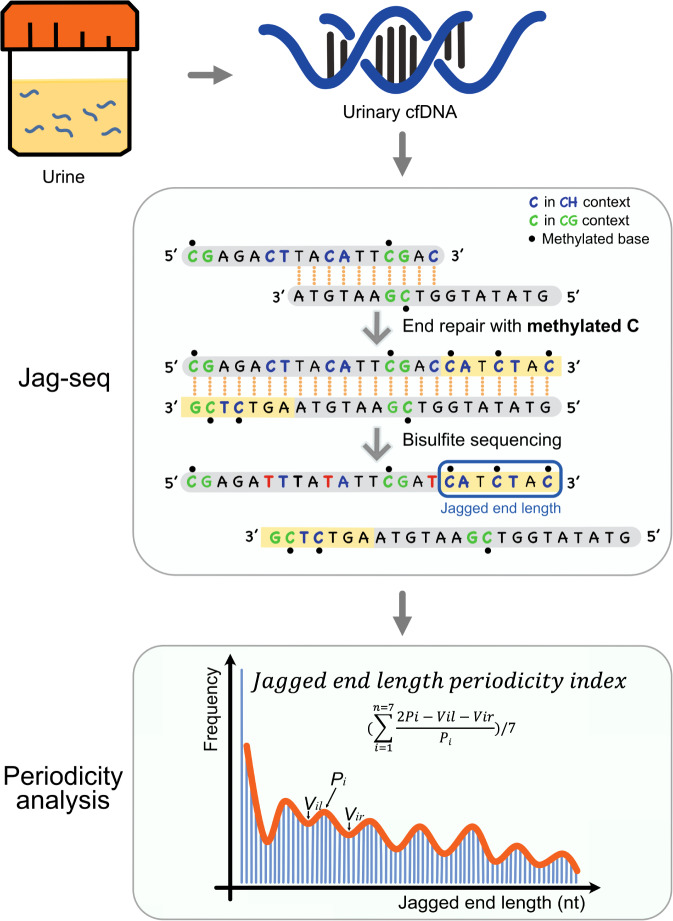
Illustration for jagged end analysis in this study. The 5′ protruding ends of urinary double-stranded molecules were filled up with dATP (A), dGTP (G), dTTP (T), and methylated dCTP (5mC) with the use of Hemo KlenTaq DNA polymerase that lacked 3′→5′ exonuclease activity and strand displacement. The methylation signals were introduced through such end-repair process. Based on bisulfite sequencing, the methylation signals at the CH sites near the 3′ end of a DNA strand indicates the presence of jagged ends. The exact jagged end length of a DNA molecule can be deduced with the two consecutive Cs in the strand with the presence of jagged ends, in which the first C is detected to be unmethylated by bisulfite treatment, but the second C be methylated. Jagged end length periodicity index was measured with series of peaks (*P*_*i*_) and troughs (*V*_*i*_) and used for renal cell carcinoma detection.

In this study, we adopted a high-resolution Jag-seq approach to investigate the properties of urinary DNA jagged ends in human subjects with and without renal cell carcinoma (RCC) (Fig. 1).

## Results

### Evaluation of modified Jag-seq based on synthetic oligonucleotides

We refined the Jag-seq method by removing the step of 3′->5′ exonuclease T (Exo T) treatment, thus preserving the authenticity of urinary DNA ends. The missing complements of 5′ single-stranded protruding DNA ends present in a double-stranded DNA molecule were restored using dATPs (As), dTTPs (Ts), dGTPs (Gs), and methylated dCTPs (i.e. mCs) using an end-repair process, resulting in blunt ends. Such end-repaired DNA molecules were ligated with sequencing adapters, followed by bisulfite treatment. Hence, the methylated signal at CH sites proximal to the ends of urinary DNA molecule could be used for reflecting the jaggedness of urinary DNA.

**Fig. 2:**
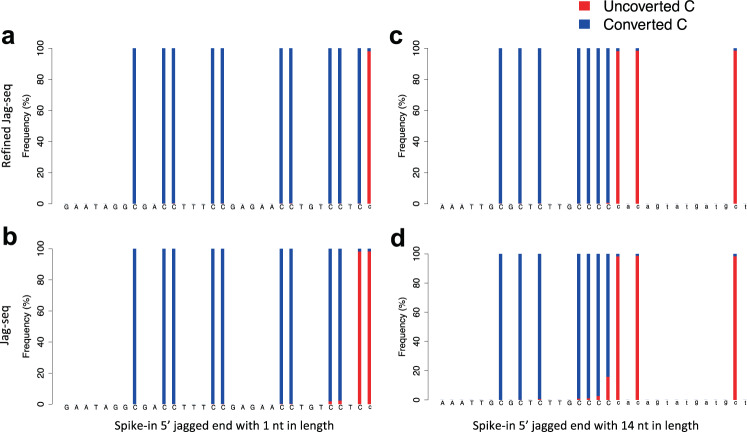
Validation for spike-in molecules with 5′ protruding jagged ends. **a**–**d** Sequencing base compositions for spike-in sequence with known jagged ends. The partial sequence of the spiked-in sequences with a 1-nt and a 14-nt jagged end are indicated on the x-axis. The nucleotides denoted in the uppercase letters indicate that the sequences are in double-stranded form. The nucleotides in lowercase letters indicate that the sequences are newly filled during the end-repair process. Vertical bars with blue color and red color represent the frequencies of sequenced T and C, respectively. Sequencing results from the refined (**a,** **c**) and original version (**b,** **d**) of Jag-seq are shown.

To validate this refined Jag-seq, we designed two synthetic oligonucleotides. The first synthetic oligonucleotide was a 46-bp molecule carrying a 1-nt 5′ protruding G nucleotide end, with another G nucleotide immediately preceding that jagged end (Supplementary Fig. 1a). The second synthetic oligonucleotide carried 14-nt 5′ protruding ends, with four consecutive C nucleotides at the recessed ends (Supplementary Fig. 1b). For data generated by an experimental protocol without Exo T (Fig. 2a), we observed that 99.9% of nucleotides at the first base relative to the 3′ end were determined to be Cs, whereas only 0.2% of nucleotides at the second base relative to the 3′ end were determined to be Cs. These results suggested that the 1-nt jagged end could be accurately detected according to the methylation signal introduced by the end-repair process. For the data generated by the previously published experimental protocol with Exo T^4^, a substantial proportion of nucleotides at the second base relative to the 3′ end was determined to be Cs (98.2%), suggesting that a proportion of 1-nt jagged ends was changed to at least 2-nt jagged ends (Fig. 2b). From the data related to the second synthetic oligonucleotide (Fig. 2d), there were 15.9% and 2.6% of nucleotides identified as Cs at the positions of 1-nt and 2-nt relative to the jagged-end starting point in data prepared by the protocol with Exo T, whereas the counterparts were nearly close to 0% (Fig. 2c). Such data suggested that Exo T would commonly introduce an extra of 1-nt jagged nucleotide in a double-stranded DNA end.

### Difference in jaggedness between plasma and urinary DNA

**Fig. 3:**
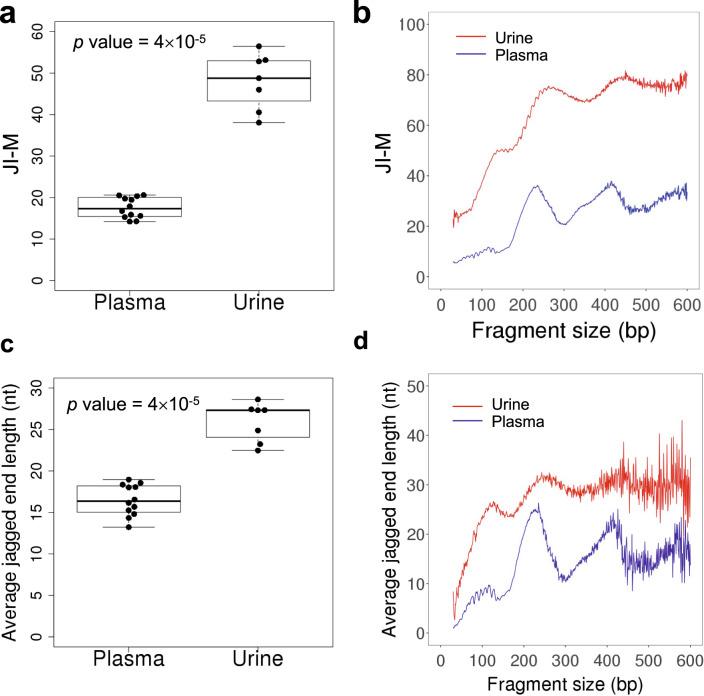
Comparison of jagged ends between plasma and urinary DNA. **a**, **b** CH methylation level in read2 (JI-M) across the different fragment sizes. **c**, **d** Average jagged end length across the different fragment sizes. The central line indicates the median value. The bottom and top of the boxes are the 25th and 75th percentiles (interquartile range). The whiskers encompass 1.5 times the interquartile range.

We applied the modified Jag-seq approach to analyze urinary DNA and plasma DNA from healthy control subjects. CH methylation levels proximal to the 3′ end of a DNA molecule (i.e. read2), which was termed Jagged index-methylated (JI-M) values, were used to reflect the jaggedness of cell-free DNA. As shown in Fig. 3a, the JI-M value of urinary DNA (median: 48.78; range: 38.08–56.47) was much higher than that in plasma DNA (median: 17.33; range: 14.22–20.62) (*p* value: 4×10^−5^, Mann–Whitney *U* test). The value of JI-M varied according to the sizes of molecules, showing wave-like patterns in both urinary and plasma DNA (Fig. 3b). For urinary DNA molecules, the JI-M value rapidly increased ranging from 25 to 80. The profile of JI-M over fragment sizes displayed a small peak at ~120 bp and a major peak at ~250 bp, followed by a second major peak at ~410 bp. We speculated that the cell-free DNA molecules with ~120 bp in size might not be associated with an intact nucleosome structure, whereas the cell-free DNA molecules at ~250 bp and ~410 bp might be associated with intact nucleosome structures. These two populations of cell-free DNA were previously reported to have different DNA nuclease accessibilities in plasma^5^. For plasma DNA, the JI-M value had a similar trend across different fragment sizes but with lower JI-M values. These results were generally consistent with the previous study^1^.

We could further determine the exact length of jagged ends for a subset of DNA molecules, on the basis of the CC-tag strategy published previously^4^. This method was to employ the methylation pattern for two consecutive cytosines in a molecule for which the first cytosine was unmethylated but the second cytosine was methylated. Such a methylation pattern enabled the determination of the starting point of a jagged end. The average jagged end length in urinary DNA (median: 27.3 nt; range: 22.5–28.6 nt) was much longer than plasma DNA (median: 16.4 nt; range: 13.2–19.0 nt) (*p* value: 4×10^−5^, Mann–Whitney *U* test) (Fig. 3c, d and Supplementary Fig. 2a, b).

### Jagged end length periodicity of urinary cfDNA

**Fig. 4:**
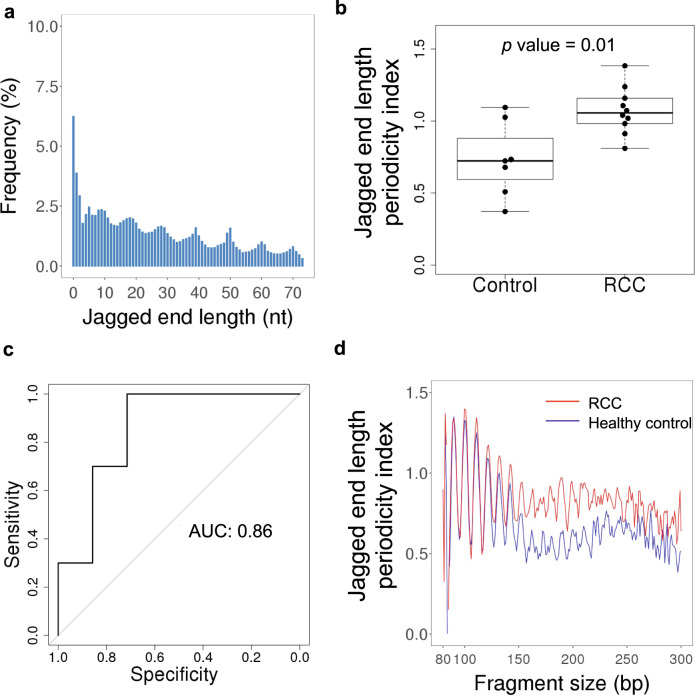
The characteristics of jagged end length of urinary DNA. **a** Jagged end length distribution measured by the CC-tag strategy. **b** Overall jagged end length periodicity index in RCC patients and healthy controls. The central line indicates the median value. The bottom and top of the boxes are the 25th and 75th percentiles (interquartile range). The whiskers encompass 1.5 times the interquartile range. **c** The area under the ROC curve (AUC) of differentiating between patients with and without RCC using jagged end length periodicity index. **d** Jagged end length periodicity index across different fragment sizes in healthy controls and patients with RCC.

We studied the relative frequency of jagged end length ranging from 0 to 74 nucleotides (nt). Generally, the longer jagged ends coincided with the lower abundance in urinary DNA (Fig. 4a). Interestingly, we observed that there were 10-nt periodic patterns of jagged end length of urinary cfDNA, which were much less observable in plasma DNA (Supplementary Fig. 3).

To quantitatively analyze the periodicity of jagged end length, we used the relative difference between a series of peak values and the paired trough values using the following formula:

1 $${\mathrm{Jagged}}\,{\mathrm{end}}\,{\mathrm{length}}\,{\mathrm{periodicity}}\,{\mathrm{index}} = \frac{{\mathop {\sum }\nolimits_{i = 1}^{n = 7} \frac{{2P_i - V_{il} - V_{ir}}}{{P_i}}}}{7}$$

Where the *P*_*i*_ is the frequency of jagged end length at a particular peak *i*, *V*_*il*_, is the frequency of jagged end length at the left valley relative to the peak *i* and *V*_*ir*_ is the frequency of jagged end length at the right valley relative to the peak *i*.

A higher jagged end length periodicity index indicated that there was a stronger 10-nt periodic patterns present in urinary DNA jagged ends. Such jagged end length periodicity index was shown to be higher in urinary DNA of patients with RCC (median: 1.06; range: 0.81–1.38), compared with control subjects (median: 0.72; range: 0.37–1.09) (*p* value: 0.01, Mann–Whitney *U* test) (Fig. 4b). The receiver operating characteristic curve (ROC) analysis showed that the area under the curve (AUC) was 0.86 in differentiating patients with and without RCC, based on the periodicity index of urinary DNA jagged end length (Fig. 4c). These results implied that the periodicity of urinary DNA jagged end length might be used as a biomarker for RCC detection.

Figure 4d shows that sharp oscillations of jagged end length periodicity index were observed in those urinary DNA molecules below 120 bp in both RCC patients and healthy controls, whereas such oscillations were attenuated for those molecules above 120 bp.

### Effects of heparin treatment on jagged end length periodicity

The strong 10-nt periodicities present in the distribution of jagged end lengths were reminiscent of the intrinsic characteristics of helical periodicity close to 10 bp per turn on the nucleosome. We conjectured that the urinary DNA molecules would be in part associated with histone proteins. As heparin with a high affinity to all histones could disrupt the interaction between DNA and histone proteins^6,7^, we used heparin to treat the urinary DNA to study whether the 10-nt periodicities of jagged end lengths would be dependent on histone proteins or not.

**Fig. 5:**
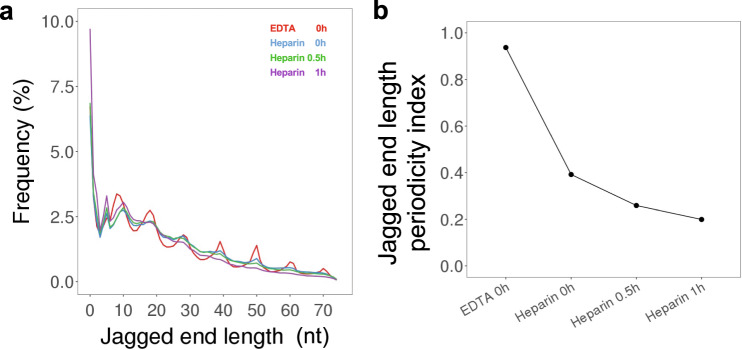
Effects of heparin treatment on jagged ends of urinary cfDNA. **a** Jagged end length distribution in urinary cfDNA with heparin incubation treatment. The lines with red, blue, green, and purple colors represent jagged end length distribution at EDTA 0 h, heparin 0 h, heparin 0.5 h, and heparin 1 h treatment, respectively. **b** Jagged end length periodicity index with EDTA and heparin treatment.

We performed EDTA treatment and heparin treatment on urine samples for 0 h, 0.5 h, and 1 h of in vitro incubation under room temperature, followed by Jag-seq. The 10-nt periodicities were remarkably reduced for the urine samples treated with heparin even at 0 h, compared with those treated by ETDA (Fig. 5a). With the prolonged periods of heparin incubation, the periodicity index was gradually decreased (Fig. 5b). Both the JI-M value and the average jagged end length showed decreased trends with heparin treatment (Supplementary Fig. 4a, b). These data suggested that the urinary DNA would be in part associated with histone proteins.

### Relationship between jagged ends and nucleosomal structures

**Fig. 6:**
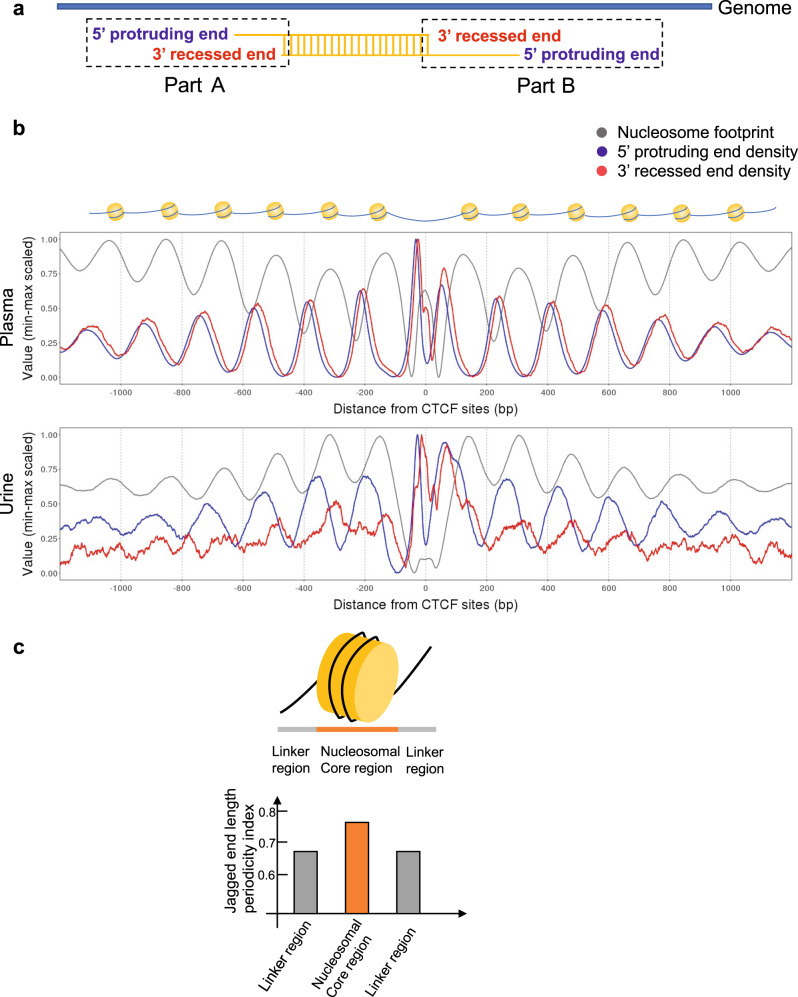
Relationship between jagged ends and nucleosome tracks. **a** Illustration for the definition of 5′ protruding end and 3′ recessed end in part A and part B. **b** 5′ protruding end density and 3′ recessed end density surrounding CTCF binding sites for plasma and urinary DNA molecules in part A. **c** Jagged end length periodicity index in nucleosomal linker regions and nucleosomal core regions.

We recently presented evidence that the fragmentation patterns of cfDNA were associated with nucleosomal structures in urine^1,7^ and plasma^8–10^. High-resolution Jag-seq could provide an opportunity to study the 3′ recessed ends and the corresponding 5′ protruding ends in the complementary strand for a cell-free DNA molecule (Fig. 6a). For a double-stranded DNA molecule with 5′ overhangs, there were two pairs of 3′ recessed ends and 5′ protruding ends, namely part A (corresponding to a lower value in the genome coordinate) and part B (corresponding to a higher value in the genome coordinate) (Fig. 6a).

We sought to investigate whether the 3′ recessed end density and the 5′ protruding end density would be related to nucleosomal structures. We applied the analysis in urinary DNA molecules falling within the 1-kb upstream and downstream relative to CTCF binding sites and calculated the end density around CTCF binding sites. The nucleosome tracks around CTCF binding sites were determined by the cell-free DNA coverages (gray lines in Fig. 6b). The preferred cutting sites during cfDNA generation were indicated by the peaks of end density^11^.

Figure 6b shows the results of plasma DNA (upper panel) and urinary DNA (lower panel) end density derived from part A. The peaks of 5′ protruding end density and 3′ recessed end density for plasma DNA were both approximately aligned to the linker regions, indicating that generation of plasma jagged ends might be derived from the cutting of the linker regions (Fig. 6b). A small shift between the 3′ recessed and 5′ protruding end density curves was observed in plasma DNA, likely because of the presence of the jagged ends (Fig. 6b). In contrast, in urine samples, the peaks of 3′ recessed end density and 5′ protruding end density were phased in different locations. The large shift between 3′ recessed and 5′ protruding end density curves indicated that the DNA degradation from the 3′ end might be more severe in urinary DNA, even extending into the nucleosomal cores. These data highlighted the potential interaction between urinary DNA and nucleosomal cores. The patterns derived from part B were consistent with that from part A (Supplementary Fig. 5).

We also studied the jagged end length periodicity index within all the nucleosomal core regions (143 bp) and among the linker regions (20 bp), respectively. The nucleosomal cores were defined by the 143-bp regions surrounding the peak signals of nucleosome tracks present in Fig. 6c. The linker regions were defined by the 20-bp regions at each side of a nucleosomal core. The jagged end length periodicity index was found to be higher at the nucleosomal core regions (0.77) compared with the linker regions (0.68) in urine (Fig. 6c). These results further highlighted that the generation of 10-nt periodicity of jagged end length might be associated with histone proteins in urine.

## Discussion

In this work, we demonstrated a high-fidelity experimental protocol for analyzing jagged ends of urinary DNA at single-base resolution. We revealed that the exonuclease T would artificially modify a proportion of jagged ends with an extra 1 nt or 2 nt, when it was used in DNA end-repair process for removing 3′ overhangs of double-stranded DNA to generate blunt-ends. The key refinement over a previously published protocol for jagged end analysis was removal of the exonuclease T (Exo T) step. Even though exonuclease T reportedly has a high activity of 3′->5′ exonuclease on single-stranded DNA, our data clearly suggested that exonuclease T would also bear a low activity of 3′ -> 5′ exonuclease on double-stranded regions.

With a high-fidelity experimental protocol for jagged end analysis, we found that the urinary DNA molecules bore much higher jaggedness than plasma DNA, which was consistent with a previous report^1^. For example, the median of average jagged end length among urinary DNA samples was 27.3 nt, whereas the counterpart among plasma DNA samples was 16.4 nt. Such consistency between studies suggested that the jagged ends were the intrinsic properties of cell-free DNA.

In addition, the jagged end analysis based on end-repair process with methylated cytosine would allow for the deduction of exact jagged end length for a subset of DNA molecules^4^. Jagged end lengths in urinary cfDNA displayed 10-nt periodic patterns which were not observable in plasma DNA. These results suggested that the mechanism in the production of jagged ends might be different between urinary DNA and plasma DNA. There are indeed a number of factors that are different between urine and plasma. For example, the DNASE1 activity was reported to be much higher in urine than in plasma^12^.

Interestingly, the amplitude of jagged end length periodicity enabled us to differentiate subjects suffering from RCC from healthy individuals. The periodicity index of jagged end length was significantly higher in patients with RCC. We achieved an AUC of 0.86 between healthy controls and patients with RCC using the value of jagged end length periodicity index. Thus, the periodicity index of jagged end length could serve as an emergent biomarker for RCC.

The 10-nt periodicities of jagged end length disappeared in the urinary DNA once heparin was added into the urine. As heparin could disrupt the interaction between DNA and histone proteins^6,7,13^, we thus speculated that urinary DNA would in part be associated with histone proteins, rendering such 10-nt periodicities of jagged end length present in urinary DNA. Seminucleosomal structures (H3_2_H4_2_) were reported to be energetically favorable during nucleosome disassembly^14^. The absence of 10-nt periodicities of jagged end length in plasma DNA might be due to the fact that the major DNA nuclease involving plasma DNA fragmentation was DNASE1L3 instead of DNASE1^5,15^. We further demonstrated that the 3′ recessed ends appeared to more often extend to nucleosomal cores in comparison with plasma DNA molecules. The jagged end length periodicity index was higher in nucleosomal cores than in linker regions. These results implied another evidence that the presence of urinary DNA jagged ends might in part interact with histone proteins. However, the determination of 3′ recessed end relied on the two consecutive cytosines in a fragment, thus only reflecting the information regarding a subset of cell-free DNA molecules. It would be interesting to verify this observation in another study using other methods such as ligation-based jagged end detection^16^.

In summary, urinary DNA was characterized with higher jaggedness in comparison with plasma DNA. The high-fidelity experimental protocol for jagged ends analysis revealed the 10-nt periodicities of jagged end length that were not realized before. The generation of jagged ends of urinary DNA might be in part associated with histone proteins. Such 10-nt periodicities of jagged end length had potential for RCC detection.

## Methods

### Human sample collection and processing

Urine samples of kidney cancer were recruited from the Department of Surgery of the Prince of Wales Hospital, Hong Kong. Healthy samples were recruited from the Department of Chemical Pathology of the Prince of Wales Hospital, Hong Kong. All subjects involved in this study gave written informed consent, and the study was approved by The Joint Chinese University of Hong Kong–Hospital Authority New Territories East Cluster Clinical Research Ethics Committee, under the Declaration of Helsinki. Collection and storage of fresh urine were followed as previously described^17^. Urinary DNA was extracted from 10 mL–30 mL cell-free urine component with the Wizard *Plus* Minipreps DNA Purification System; (Promega). 15 mL of 6 mol/L guanidine thiocyanate (Sigma–Aldrich) and 1 mL of resin (Wizard *Plus* Minipreps DNA Purification System; Promega) were added in each 10 mL of the processed urine. The mixture was incubated and shaken gently at room temperature for 2 h. Then, the DNA was purified and eluted into 100 μL RNase-free water using the minicolumns and according to the manufacturer’s instruction of the purification system. The quantity of urinary DNA obtained ranged from ~10 ng to ~100 ng according to Qubit assays.

### Library preparation

Hemo KlenTaq (NEB) and dATP (A), dGTP (G), dTTP (T), and methylated dCTP (5mC) were used for filling in 5′ protruding ends of double-stranded DNA molecules, forming blunt-end molecules. 3′ A-tailing on blunt ends was processed by Hemo KlenTaq. The end-repaired DNA was subjected to DNA purification using a MinElute Reaction Cleanup kit (QIAGEN). We further applied polynucleotide kinase (PNK) (NEB) for 5′ phosphorylation of purified DNA, followed by methylated adapter ligation by T4 DNA ligase (NEB), obtaining the resultant DNA libraries ready for sequencing. All methylated adapters were TruSeq single indexed adapter (Illumina). The molecules with 3′ protruding ends were not able to be ligated with sequencing adapters, thus would not be present in the sequencing results.

We treated the adapter-ligated DNA with two rounds of bisulfite conversion by EpiTect Plus DNA Bisulfite kit (Qiagen). We amplified bisulfite-converted DNA molecules with 12 cycles using KAPA HiFi HotStart Uracil + ReadyMix (Roche). PCR primers included P5 PCR Primer (5′ AATGATACGGCGACCACCGAG) and P7 PCR Primer (5′ CAAGCAGAAGACGGCATACGAG).

We examined the quality of DNA library and the size of DNA by an Agilent 4200 TapeStation with HSD1000 ScreenTape (Agilent). The DNA libraries were sequenced on a NextSeq platform (Illumina) using the paired-end mode (75-bp × 2).

### Sequencing data analysis

The adapter sequences and low-quality bases were trimmed off from the raw sequencing reads using Trim Galore^18^. The pre-processed reads were aligned to the human genome (hg38) using Bismark^19^. The median number of aligned paired-end sequencing reads was 24.1 million (interquartile range: 16.0 – 28.1 million). The jagged ends detection was performed as described in our previous study^4^. The Mann–Whitney test was used to compare the difference between groups. *P* value of <0.05 was considered statistically significant.

### Reporting summary

Further information on research design is available in the Nature Research Reporting Summary linked to this article.

## Supplementary information

supplementary data

Reporting Summary

## Data Availability

The sequence data generated and analyzed have been deposited in the European Genome-Phenome Archive (EGA), www.ebi.ac.uk/ega, hosted by the European Bioinformatics Institute (EBI), www.ebi.ac.uk (accession no. https://ega-archive.org/studies/EGAS00001005603).
