# Supplementary material for: High-resolution analysis for urinary DNA jagged ends
Source: NPJ Genom Med. 2022 Feb 23;7:14. doi: 10.1038/s41525-022-00285-1 (PMC8866458; doi:10.1038/s41525-022-00285-1)
Supplement: Supplementary file 1 — supplementary data [file 41525_2022_285_MOESM1_ESM.pdf]

Supplementary data

a

```
5′  GGTACTCAAAGAATAGGCGACCTTTCCGAGAACCTGTCCTC   3′
3′  CCATGAGTTTCTTATCCGCTGGAAAGGCTCTTGGACAGGAGG  5′
```

Spike-in 5′ jagged end with 1 nt in length

b

```
5′  TCACATTGCTAGCCAAATTGCGCTCTTGCCCC                3′
3′  AGTGTAACGATCGGTTTAACGCGAGAACGGGGGTGTCATACTACGA  5′
```

Spike-in 5′ jagged end with 14 nt in length

Supplementary Fig. 1 Spike-in molecules with 5' protruding jagged end. The sequence structure of a 1-nt (**a**) and a 14-nt (**b**) spike-in jagged end. The letters in bold red represent the 5' protruding ends.

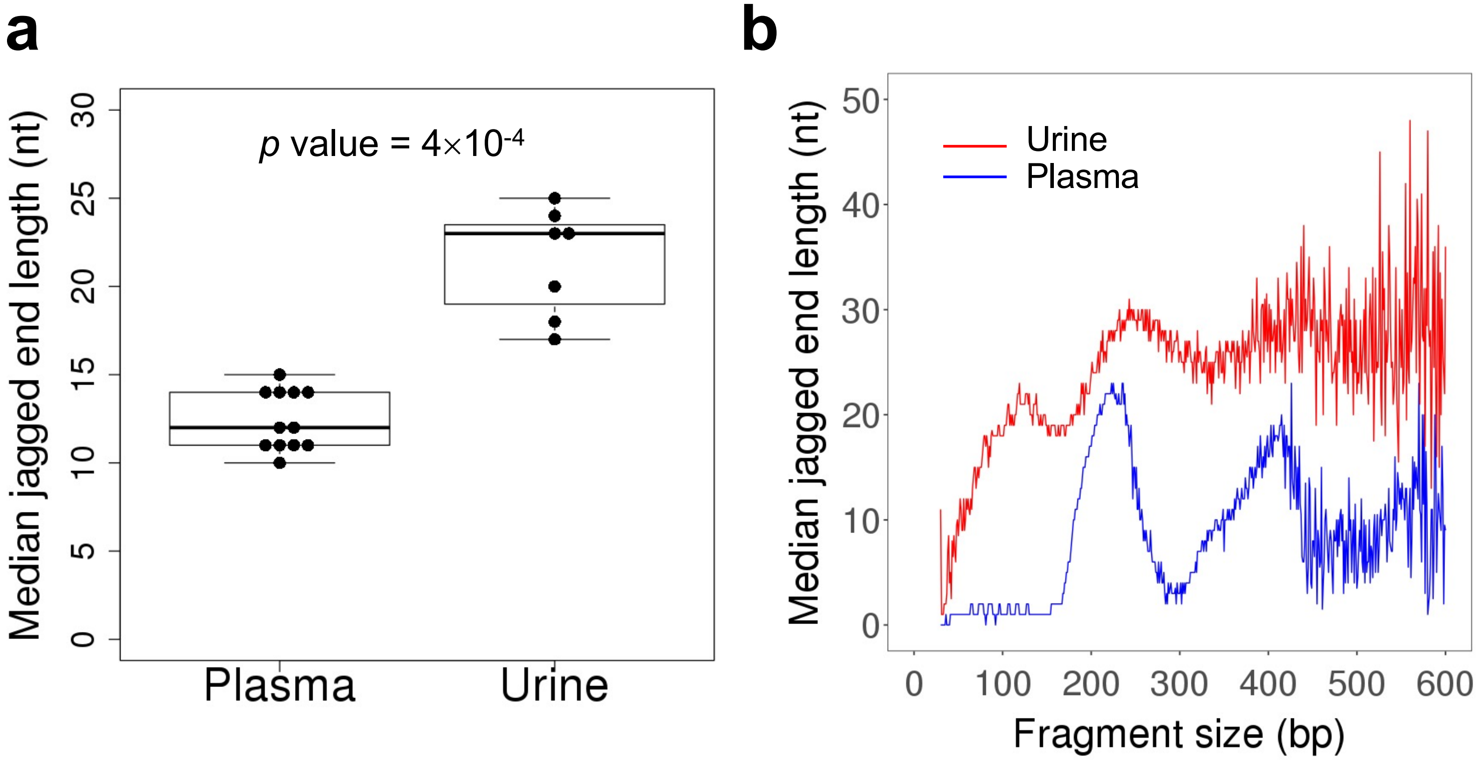

Supplementary Fig. 2 Comparison of median jagged end length between plasma and urinary DNA.

Comparison of overall median jagged end length (**a**) and median jagged end length across the different fragment sizes (**b**) between plasma and urinary DNA.

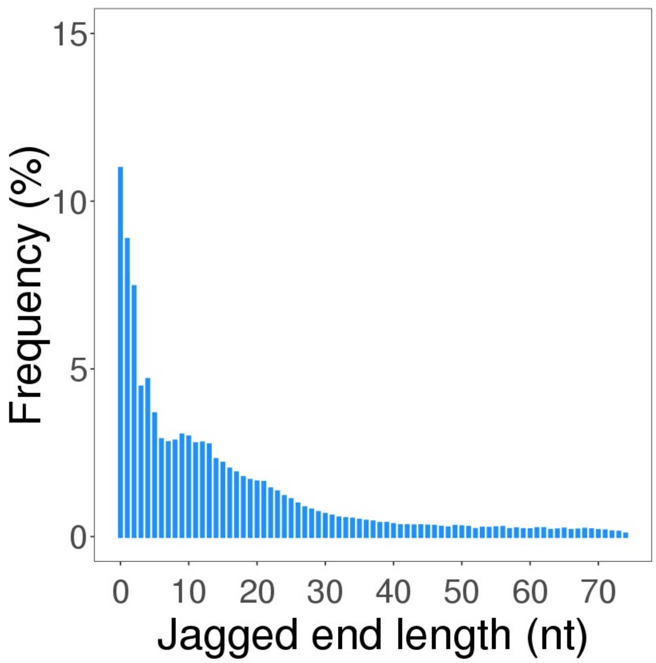

Supplementary Fig. 3 The jagged end length distribution of plasma DNA without exonuclease T (exo T) treatment.

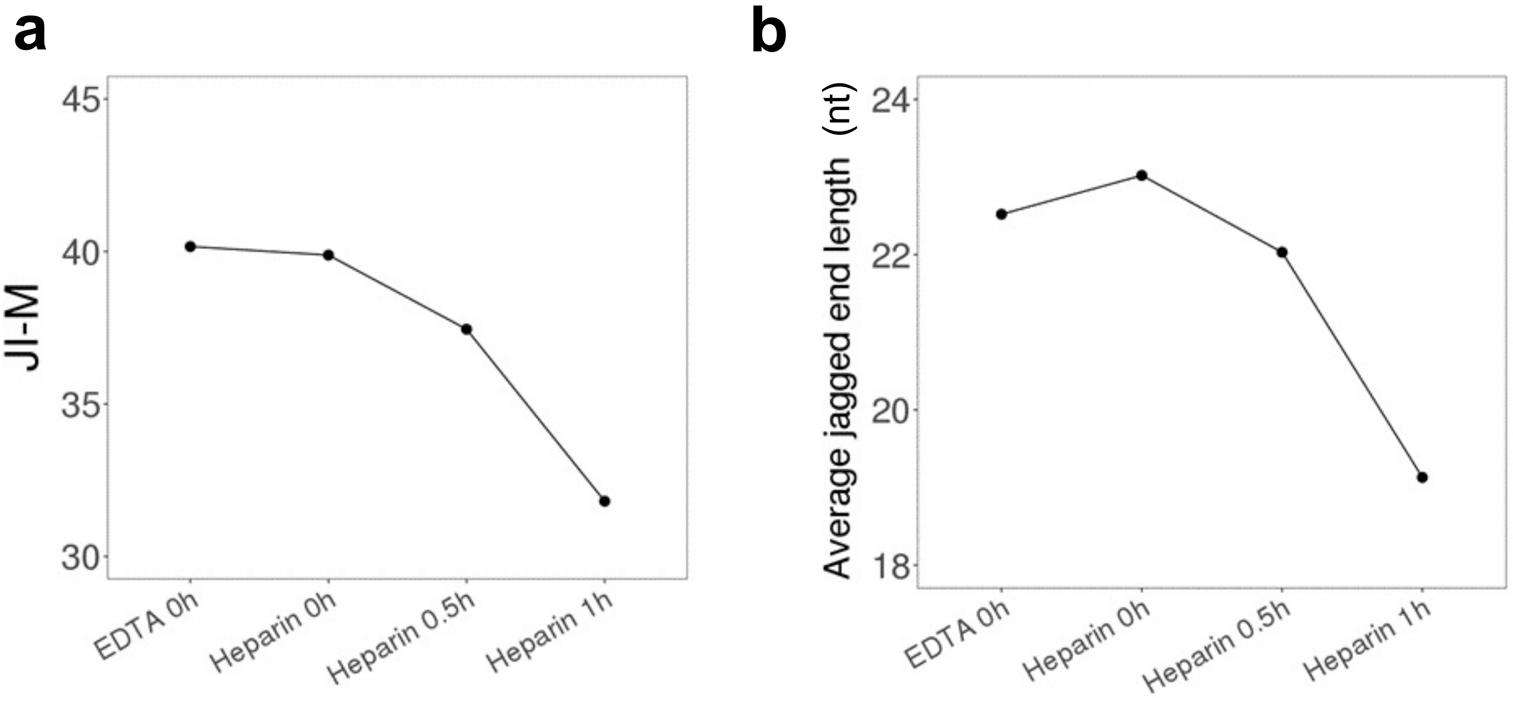

Supplementary Fig. 4 Jaggedness of urinary cfDNA with heparin incubation treatment. JI-M (**a**) and average jagged end length (**b**) of urinary cfDNA with heparin treatment over different incubation times. The lines with red, blue, green, and purple colors represented EDTA 0 h, heparin 0 h, heparin 0.5 h, and heparin 1 h treatment, respectively.

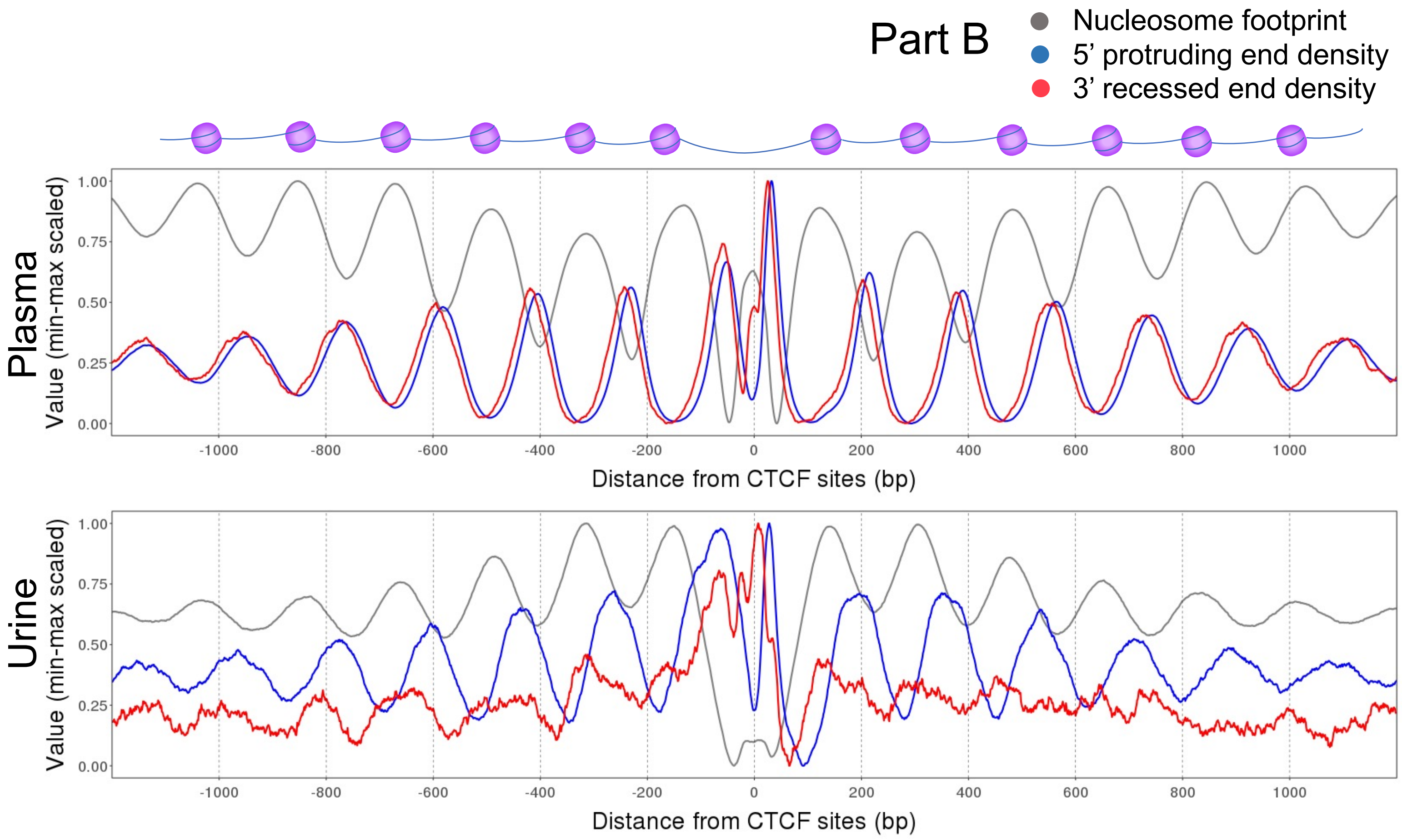

Supplementary Fig. 5 Relationship between jagged ends and nucleosome tracks. 5' protruding end density and 3'recessed end density surrounding CTCF binding sites for plasma and urinary DNA molecules in part B**.**
