# Supplementary material for: High-resolution analysis for urinary DNA jagged ends
Source: NPJ Genom Med. 2022 Feb 23;7:14. doi: 10.1038/s41525-022-00285-1 (PMC8866458; doi:10.1038/s41525-022-00285-1)
Supplement: Supplementary file 2 — Reporting Summary [file 41525_2022_285_MOESM2_ESM.pdf]

nature portfolio

Corresponding author(s): LYMD

Last updated by author(s): 2021-11.29

Reporting Summary

Nature Portfolio wishes to improve the reproducibility of the work that we publish. This form provides structure for consistency and transparency in reporting. For further information on Nature Portfolio policies, see our Editorial Policies and the Editorial Policy Checklist.

Statistics

For all statistical analyses, confirm that the following items are present in the figure legend, table legend, main text, or Methods section.

n/a	Confirmed	
☐	☒	The exact sample size (*n*) for each experimental group/condition, given as a discrete number and unit of measurement
☐	☒	A statement on whether measurements were taken from distinct samples or whether the same sample was measured repeatedly
☐	☒	The statistical test(s) used AND whether they are one- or two-sided<br>*Only common tests should be described solely by name; describe more complex techniques in the Methods section.*
☐	☒	A description of all covariates tested
☒	☐	A description of any assumptions or corrections, such as tests of normality and adjustment for multiple comparisons
☐	☒	A full description of the statistical parameters including central tendency (e.g. means) or other basic estimates (e.g. regression coefficient) AND variation (e.g. standard deviation) or associated estimates of uncertainty (e.g. confidence intervals)
☐	☒	For null hypothesis testing, the test statistic (e.g. *F*, *t*, *r*) with confidence intervals, effect sizes, degrees of freedom and *P* value noted<br>*Give P values as exact values whenever suitable.*
☒	☐	For Bayesian analysis, information on the choice of priors and Markov chain Monte Carlo settings
☒	☐	For hierarchical and complex designs, identification of the appropriate level for tests and full reporting of outcomes
☒	☐	Estimates of effect sizes (e.g. Cohen's *d*, Pearson's *r*), indicating how they were calculated

Our web collection on statistics for biologists contains articles on many of the points above.

Software and code

Policy information about availability of computer code

Data collection	No software was used
Data analysis	Bismark (Version: v0.22.3, https://github.com/FelixKrueger/Bismark) was used for performing the alignment of bisulfite sequencing. Python (version: 3.7.4, https://www.python.org) was used for anazlying the jagged ends of cell-free DNA. R (version: 3.6.2, https://www.r-project.org) was used for performing statistics analysis.

For manuscripts utilizing custom algorithms or software that are central to the research but not yet described in published literature, software must be made available to editors and reviewers. We strongly encourage code deposition in a community repository (e.g. GitHub). See the Nature Portfolio guidelines for submitting code & software for further information.

Data

Policy information about availability of data

All manuscripts must include a data availability statement. This statement should provide the following information, where applicable:

- Accession codes, unique identifiers, or web links for publicly available datasets
- A description of any restrictions on data availability
- For clinical datasets or third party data, please ensure that the statement adheres to our policy

The sequence data generated and analyzed have been deposited in the European Genome-Phenome Archive (EGA), www.ebi.ac.uk/ega, hosted by the European Bioinformatics Institute (EBI), www.ebi.ac.uk (accession no. https://ega-archive.org/studies/EGA S00001005603).

Field-specific reporting

Please select the one below that is the best fit for your research. If you are not sure, read the appropriate sections before making your selection.

☒ Life sciences ☐ Behavioural & social sciences ☐ Ecological, evolutionary & environmental sciences

For a reference copy of the document with all sections, see nature.com/documents/nr-reporting-summary-flat.pdf

Life sciences study design

All studies must disclose on these points even when the disclosure is negative.

Sample size	In this exploratory study, Mann-Whitney U test was used for testing whether a difference observed between two groups would be statistically significant. Mann-Whitney U test would require at least 4 samples for each group to discern the difference, if present, with a statistical significance (p value < 0.05). We collected at least 7 samples for each group for the data present in Figure 3a, Figure 3c and Figure 4b which involved Mann-Whitney U test. The observed difference in these three figures were shown to be statistically significant, with P values of 0.00004, 0.00004 and 0.01, respectively. Thus, the sample sizes used in this exploratory study should be sufficient to support conclusions stated in the manuscript.
Data exclusions	No data were excluded from the analyses.
Replication	The refined approach for jagged end analysis of cell-free DNA present in this study showed the consistent result with the previously-published study based on the different approach and different sample cohort (Zhou et al. Clin Chem. 2021;67:621-630), i.e. urinary DNA molecules contained much higher jaggedness than plasma DNA molecules.
Randomization	Samples were randomly allocated into different experimental groups.
Blinding	Blinding was not relevant to our study. The current study attempted to explore an approach for high-resolution jagged end analysis of urinary DNA. Jagged end properties were compared between the urinary and plasma DNA molecules, as well as between urinary DNA samples of human subjects with and without renal cell carcinoma. Thus, the sample information was made known beforehand.

Reporting for specific materials, systems and methods

We require information from authors about some types of materials, experimental systems and methods used in many studies. Here, indicate whether each material, system or method listed is relevant to your study. If you are not sure if a list item applies to your research, read the appropriate section before selecting a response.

Materials & experimental systems

n/a	Involved in the study	
☒	☐	Antibodies
☒	☐	Eukaryotic cell lines
☒	☐	Palaeontology and archaeology
☒	☐	Animals and other organisms
☐	☒	Human research participants
☒	☐	Clinical data
☒	☐	Dual use research of concern

Methods

n/a	Involved in the study	
☒	☐	ChIP-seq
☒	☐	Flow cytometry
☒	☐	MRI-based neuroimaging

Human research participants

Policy information about studies involving human research participants

Population characteristics	The median age of subjects used in this study was 56 years (range: 25-72 years). There were 35% and 65% of female and male subjects, respectively.
Recruitment	Urine samples of kidney cancer were recruited from the Department of Surgery of the Prince of Wales Hospital, Hong Kong. Healthy samples were recruited from the Department of Chemical Pathology of the Prince of Wales Hospital, Hong Kong.
Ethics oversight	The Joint Chinese University of Hong Kong–Hospital Authority New Territories East Cluster Clinical Research Ethics Committee, under the Declaration of Helsinki.

Note that full information on the approval of the study protocol must also be provided in the manuscript.
